# Simon’s Manual of Chemistry

**Published:** 1905-12

**Authors:** 


SIMON'S MANUAL OF CHEMISTRY. A Guide to Lectures and Laboratory work for beginners in chemistry. A text-book especially adapted for students of medicine, pharmacy and dentistry. By William Simon, Ph.D., M.D., Professor of Chemistry in the College of Physicians and Surgeons of Baltimore, and in the Baltimore College of Dental Surgery, etc. New (8th) edition, thoroughly revised to conform with the eighth decennial revision of the U. S. Pharmacopœia. In one octavo volume of 643 pages with 66 engravings, 8 colored plates representing 64 important chemical reactions, and one colored spectra plate. Cloth, $3.00 net. Lea Brothers & Co., Publishers, Philadelphia and New York, 1905.

This well-known book was excellent from the beginning, but now the eighth edition with its revisions is one of our most valuable manuals on chemistry. It comprises all the important facts in chemical physics, inorganic, organic, analytical and physiological chemistry, and is written in a style that is clear and comprehensive.

The changes and additions to the new Pharmacopœia, that are of chemiaal interest, have been incorporated in this edition.

The importance of physiological chemistry can not be overestimated and no progressive physician can afford to lag behind in this branch, for in it many radical changes have been made in recent years, and especially on account of its close relation to immunity, etc.

